# Vasopressin Versus Norepinephrine for the Management of Shock After Cardiac Surgery (VaNCS study): a randomized controlled trial

**DOI:** 10.1186/cc12160

**Published:** 2013-03-19

**Authors:** L Hajjar, JL Vincent, A Rhodes, D Annane, F Galas, J Almeida, S Zeferino, L Camara, V Santos, J Pereira, E Osawa, E Maciel, A Rodrigues, J Jardim, D Blini, E Araujo, F Bergamin, R Kalil Filho, J Auler Jr

**Affiliations:** 1 Heart Institute, São Paulo, Brazil; 2 Universite Libre de Bruxelles, Brussels, Belgium; 3 University of London, UK; 4 Faculte Medicine Paris, France; 5 Cancer Institute, São Paulo, Brazil

## Introduction

Vasoplegic syndrome is a common complication after cardiac surgery, with negative impact on patient outcomes and hospital costs. Pathogenesis of vasodilatory phenomenon after cardiac surgery remains a matter of controversy. Loss of vascular tone can be partly explained by the depletion of neurohypophyseal arginine vasopressin stores. Vasopressin is commonly used as an adjunct to catecholamines to support blood pressure in refractory septic shock, but its effect on vasoplegic shock is unknown. We hypothesized that the use of vasopressin would be more effective on treatment of shock after cardiac surgery than norepinephrine, decreasing the composite endpoint of mortality and severe morbidity.

## Methods

In this prospective and randomized, double-blind trial, we assigned patients who had vasoplegic shock to receive either vasopressin (0.01 to 0.06 U/minute) or norepinephrine (0.01 to 1 μg/ kg/minute) in addition to open-label vasopressors. All vasopressor infusions were titrated and tapered according to protocols to maintain a target blood pressure. The primary endpoint was major morbidity according to STS (30-day mortality, mechanical ventilation >48 hours, mediastinitis, surgical re-exploration, stroke, acute renal failure). Secondary outcomes were time on mechanical ventilation, ICU and hospital stay, new infection, the time to attainment of hemodynamic stability, occurrence of adverse events and safety.

## Results

A total of 300 patients underwent randomization, were infused with the study drug (148 patients received vasopressin, and 152 norepinephrine), and were included in the analysis. Patients who received vasopressin had a lower rate of morbidity (23.5% vs. 34%, *P *= 0.001) as compared with the norepinephrine group. The 30-day mortality rate was 6.1% in the norepinephrine group and 4.6% in the vasopressin group (*P *= 0.570). There were no significant differences in the overall rates of serious adverse events (7.4% and 6.6%, respectively; *P *= 0.772).

## Conclusion

Vasopressin reduces major morbidity after cardiac surgery as compared with norepinephrine among patients with cardiac surgery with vasoplegic shock.

